# Hyperviscosity Syndrome, Hypercalcemia, and Acute Renal Failure as Initial Manifestations of Multiple Myeloma in an Octogenarian

**DOI:** 10.1155/crh/1910259

**Published:** 2026-07-29

**Authors:** Wen-Chun Yu, Hui-Yun Chiu, Hung-Bin Tsai

**Affiliations:** ^1^ Department of Nursing, National Taiwan University Hospital, Taipei, Taiwan, ntuh.gov.tw; ^2^ Division of Hospital Medicine, Department of Internal Medicine, National Taiwan University Hospital, Taipei, Taiwan, ntuh.gov.tw; ^3^ Division of Hospital Medicine, Department of Internal Medicine, Taipei City Hospital Zhongxing Branch, Taipei, Taiwan, taipei.gov.tw

**Keywords:** hypercalcemia, hyperviscosity syndrome, multiple myeloma

## Abstract

**Background:**

Multiple myeloma (MM) is a hematological malignancy characterized by the proliferation of abnormal plasma cells, often accompanied by the excessive secretion of monoclonal immunoglobulins. Hyperviscosity syndrome (HVS) is a rare but life‐threatening complication that leads to microcirculatory impairment and organ dysfunction. Additionally, hypercalcemia, commonly associated with osteolytic bone destruction, can further result in acute kidney injury and altered consciousness.

**Case Presentation:**

We describe an 80‐year‐old male who presented with confusion, generalized weakness, decreased urine output, and fever. He was subsequently diagnosed with MM complicated by HVS, hypercalcemia, and acute renal failure. Initial investigations revealed severe anemia, significantly elevated IgG levels (8612.26 mg/dL), multiple osteolytic lesions, and deteriorating renal function. The patient also exhibited epistaxis and neurological symptoms. Emergency plasmapheresis was initiated, alongside calcitonin and supportive therapy for hypercalcemia. Bone marrow examination confirmed plasma cell myeloma. Following five sessions of plasmapheresis, immunoglobulin levels decreased significantly, and clinical symptoms improved markedly. The patient was then successfully transitioned to immunomodulatory agents and systemic chemotherapy.

**Conclusion:**

MM presenting with HVS and hypercalcemia can be rapidly fatal. Early recognition and immediate intervention with plasmapheresis and supportive care are crucial for improving patient outcomes and facilitating subsequent oncological treatment.

## 1. Introduction

Multiple myeloma (MM) is a hematological malignancy arising from the malignant proliferation of plasma cells in the bone marrow. It is characterized by the secretion of monoclonal immunoglobulins, osteolytic bone destruction, anemia, hypercalcemia, and renal insufficiency [1, 2]. As the disease progresses, some patients develop acute, life‐threatening complications. Among these, hyperviscosity syndrome (HVS) is relatively rare but carries a high mortality rate, necessitating rapid diagnosis and intervention [3, 4].

HVS results from an abnormal increase in serum immunoglobulin levels, which elevates blood viscosity and leads to microvascular circulatory impairment and tissue hypoperfusion. Patients may present with blurred vision, altered mental status, neurological abnormalities, and mucosal bleeding [3, 5]. While HVS is most commonly associated with IgM‐type Waldenström’s macroglobulinemia (WM), it can occur in MM when IgG levels are profoundly elevated [3, 4]. Furthermore, hypercalcemia is a frequent complication of MM, typically linked to osteolysis, and in severe cases, it can lead to dehydration, acute kidney injury (AKI), and confusion [6, 7].

Current clinical guidelines recommend that when MM is complicated by HVS or acute organ injury, emergency supportive care should be initiated concurrently with antimyeloma therapy [1, 8, 9]. Plasmapheresis is the first‐line emergency treatment for HVS as it rapidly reduces the concentration of abnormal immunoglobulins [3]. Hypercalcemia requires aggressive management through hydration, calcitonin, and other calcium‐lowering agents [7]. This report describes an elderly patient presenting with HVS, hypercalcemia, and acute renal failure as the initial manifestations of MM and discusses the diagnostic process, acute management, and subsequent treatment strategies.

## 2. Case Presentation

### 2.1. Patient Information

An 80‐year‐old male with a history of hypertension, Stage 3 chronic kidney disease (CKD), and chronic glomerulonephritis presented to the emergency department. The patient was previously independent in daily activities but had experienced progressive weakness and decreased activity over the past month, including a fall at home. He subsequently developed fever, confusion, and oliguria, leading to hospitalization.

## 3. Clinical Examination and Diagnosis

**FIGURE 1:**
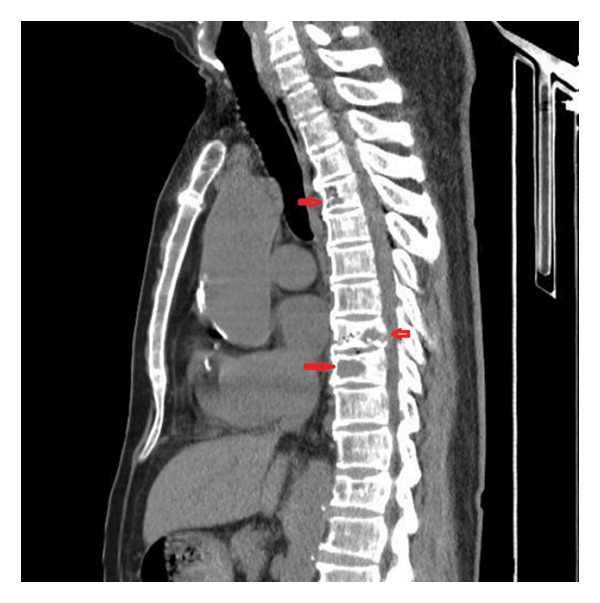
Computed tomography showing multiple osteolytic lesions.

Upon admission, a whole‐body computed tomography (CT) scan revealed multiple osteolytic lesions (Figure 1), raising suspicion of metastatic malignancy. Laboratory findings showed an elevated white blood cell count (10.08 k/μL) and C‐reactive protein (11.33 mg/dL). A peripheral blood smear identified approximately 16% immature, suspicious plasma cells. Hypercalcemia was also noted (3.04 mmol/L).

**FIGURE 2:**
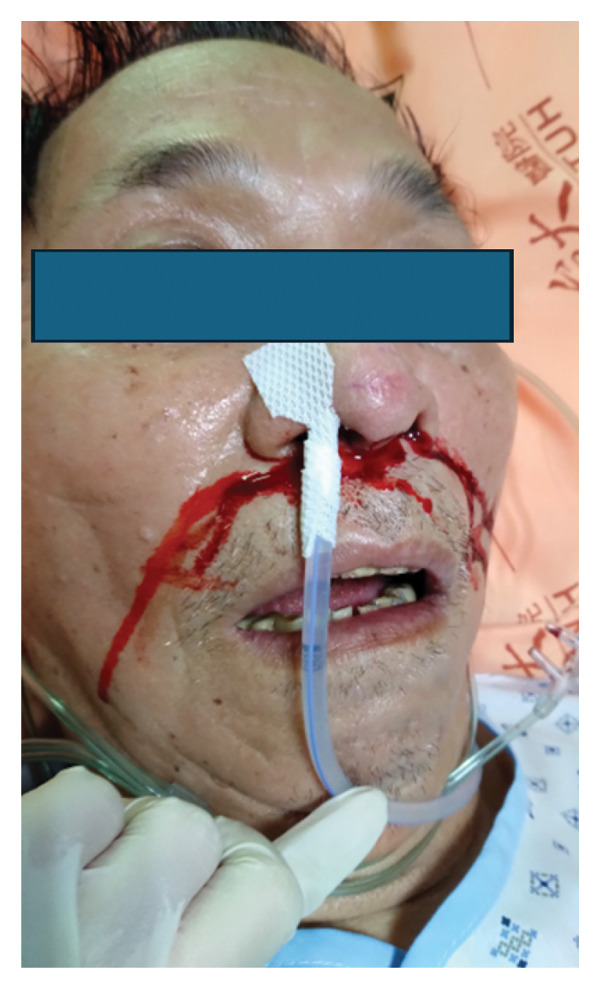
Epistaxis.

During hospitalization, the patient received antibiotics for suspected pneumonia. However, subsequent laboratory tests revealed severe anemia (Hemoglobin 2.1 g/dL), hypernatremia (147 mmol/L), and rapidly declining renal function (BUN 85.5 mg/dL, creatinine 3.3 mg/dL). Further analysis showed an inverted albumin‐to‐globulin ratio (albumin 1.9 g/dL, total protein 11.2 g/dL), with IgG significantly elevated at 8612.26 mg/dL. Parathyroid hormone (PTH) levels were normal (41 pg/mL). Fluorescence in situ hybridization (FISH) did not detect high‐risk cytogenetic abnormalities such as del(17p) [1, 2]. The patient also experienced recurrent epistaxis (Figure 2) and fluctuating levels of consciousness. Based on the clinical presentation and laboratory results, HVS was highly suspected [3, 4].

### 3.1. Treatment and Outcomes

**FIGURE 3:**
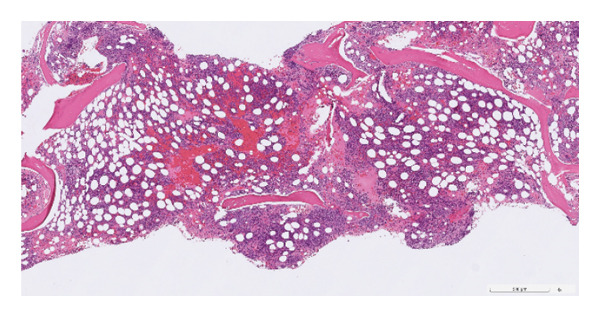
Bone marrow biopsy (H&E stain, 4x magnification) showing a hypercellular marrow with extensive infiltration, where normal hematopoietic elements are largely replaced by abnormal cells.

**FIGURE 4:**
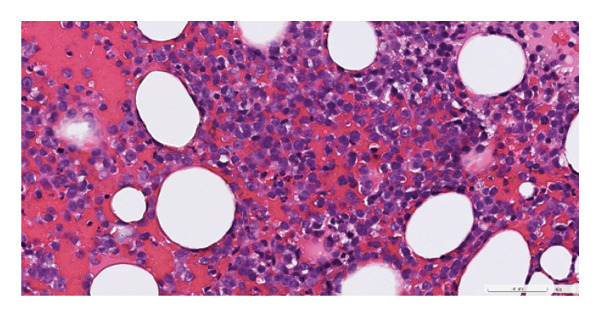
Higher magnification of the bone marrow biopsy (H&E stain, 40x magnification) revealing sheets of immature and atypical plasma cells with eccentric nuclei.

**FIGURE 5:**
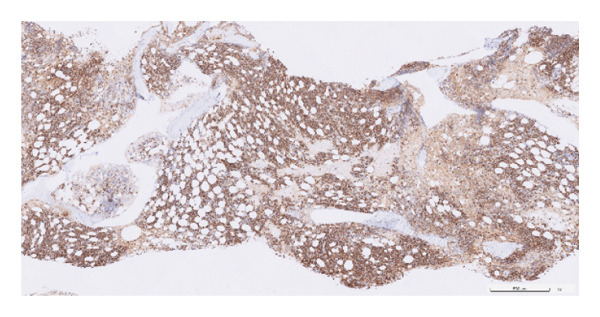
Immunohistochemical staining (10x magnification) demonstrating strong positive expression of CD56 in the infiltrating abnormal plasma cells.

**FIGURE 6:**
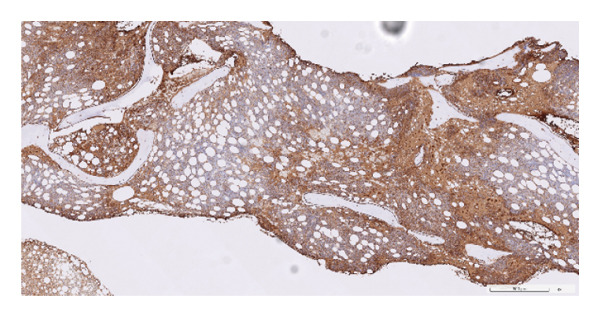
Immunohistochemical staining (4x magnification) showing diffuse strong cytoplasmic positivity for lambda (λ) light chain, indicating lambda light chain restriction and monoclonal proliferation.

Given the hyper‐IgG‐emia, epistaxis, and neurological symptoms, the medical team immediately initiated plasmapheresis to reduce blood viscosity [3, 4]. Concurrently, calcitonin and supportive fluid therapy were administered to control hypercalcemia [7]. Subsequent bone marrow aspiration and biopsy revealed that over 90% of cells were immature plasma cells (Figures 3 and 4). Immunohistochemistry was positive for CD138 with lambda light chain restriction (Figures 5 and 6), confirming the diagnosis of plasma cell myeloma.

Following the diagnosis, the patient was initially treated with the immunomodulatory agent thalidomide. After five sessions of plasmapheresis, the IgG level dropped significantly (8612.26 mg/dL to 1156.95 mg/dL), and clinical symptoms improved markedly. The patient was subsequently transferred to the hematology department to initiate the VTD regimen (bortezomib, thalidomide, and dexamethasone). His condition stabilized, and he was discharged with scheduled outpatient follow‐ups.

## 4. Discussion

This case involves an elderly patient with MM presenting with life‐threatening complications, including HVS, hypercalcemia, and acute renal failure. Because the initial symptoms—confusion, fever, and renal impairment—are nonspecific, they are often attributed to infection, sepsis, or other metastatic malignancies, which increases the difficulty of early MM recognition.

In the differential diagnosis of HVS, it is essential to distinguish between MM and WM. While WM is typically caused by IgM overproduction, this case was driven by an extremely high concentration of IgG. Although IgG‐type HVS is less common, significantly elevated levels can still lead to microcirculatory obstruction and organ hypoperfusion [3, 4]. The epistaxis, altered consciousness, and renal deterioration observed in this patient are hallmark clinical signs of HVS [5, 10].

Furthermore, the patient presented with concurrent hypercalcemia and AKI. In MM, hypercalcemia primarily results from increased osteoclast activity and bone destruction, which can lead to dehydration and neurological deficits [6, 7]. In this case, the normal PTH value helped exclude primary hyperparathyroidism, and the bone marrow examination confirmed MM as the underlying etiology [9].

International guidelines suggest that if MM is associated with HVS or acute organ injury, emergency supportive measures and disease‐specific therapy should be initiated simultaneously [1, 8]. Plasmapheresis remains the most critical emergency intervention for HVS as it rapidly removes abnormal immunoglobulins [3]. The significant IgG reduction and clinical improvement following five sessions in this case underscore the importance of prompt intervention. For subsequent therapy, considering the patient’s age and renal impairment, thalidomide was chosen for initial modulation, followed by the proteasome inhibitor–based VTD regimen [1, 2]. This strategy successfully suppressed the malignant plasma cells and stabilized the disease.

Notably, there were limitations in imaging. The initial evaluation used standard CT, which quickly identified lytic lesions but is less sensitive to early bone marrow infiltration compared to whole‐body low‐dose CT (WBLDCT), MRI, or FDG PET‐CT [11]. Thus, the actual extent of bone marrow involvement may have been underestimated.

## 5. Conclusion

This case demonstrates that MM can present acutely with life‐threatening HVS, hypercalcemia, and renal failure. Clinicians should maintain a high index of suspicion for MM and HVS in elderly patients presenting with unexplained altered consciousness, mucosal bleeding, severe anemia, and hyperproteinemia. Plasmapheresis is the cornerstone of emergency treatment for HVS. Early management of complications and the introduction of antimyeloma therapy are vital for improving prognosis [3, 7, 10].

## Funding

No funding was received for this manuscript.

## Ethics Statement

This case report was conducted in accordance with the principles outlined in the Declaration of Helsinki. All identifying information has been removed to protect patient confidentiality. Informed consent was obtained from the patient for publication of this case and any accompanying images. Institutional review board approval was not required.

## Conflicts of Interest

The authors declare no conflicts of interest.

## Data Availability

The data that support the findings of this study are openly available in (Wen‐Chun Yu) at (https://orcid.org/0009-0002-7445-6724).
